# Knowledge, attitude and practice towards cervical cancer prevention among mothers of girls aged between 9 and 14 years: a cross sectional survey in Zimbabwe

**DOI:** 10.1186/s12905-021-01575-z

**Published:** 2021-12-20

**Authors:** Petmore Zibako, Nomsa Tsikai, Sarah Manyame, Themba G. Ginindza

**Affiliations:** 1 Discipline of Public Health Medicine, School of Nursing and Public Health, University of KwaZulu-Natal, 2nd Floor George Campbell Building, Howard College Campus, Durban, 4041 South Africa; 2 College of Health Sciences, University of Zimbabwe, MP167 Harare, Zimbabwe

**Keywords:** Cervical cancer prevention, Human papillomavirus vaccine, Cervical cancer screening, Knowledge, Attitude, Practice, Human papillimavirus

## Abstract

**Background:**

Cervical cancer (CC) morbidity and mortality is still high in developing countries like Zimbabwe. Treatment for CC is out of reach for many women, hence the need to maximise on prevention which mainly includes screening and administering human papillomavirus (HPV) vaccine. Knowledge about CC prevention is a prerequisite for utilisation of all the available options for CC prevention, yet little is known about its levels and the corresponding attitudes and practices on cancer prevention methods within the society.

**Methods:**

A cross sectional survey was done to assess knowledge, attitude and practice (KAP) on CC prevention among mothers of girls aged between 9 and 14 years in Zimbabwe as well as factors explaining the KAP. Four hundred and six mothers participate. Descriptive and inferential statistics (binary logistic regression and Chi-Square test of association) were applied to determine participant characteristics with KAP using STATA version 16 software.

**Findings:**

Overall KAP of cervical cancer prevention is in a poor state. The knowledge was poor with 24% being able to say CC is caused by HPV; the attitude is negative with 58% being of the opinion that CC is caused by witchcraft and it is a death sentence, while the bad practices of relying only on traditional means were being practiced. Factors associated with knowledge are: not having medical aid (odds: 0.17, 95%CI: 0.05–0.59, *p* = 0.005) and high levels of education (secondary level odds: 4.20; 95%CI: 2.25–7.84 *p* < 0.001 and tertiary odds: 7.75; 95%CI: 2.04–29.45, *p*-value: 0.003 compared to primary education). Attitude towards CC management was driven by levels of education (secondary level odds: 0.39, 95%CI: 0.20–0.78, *p* = 0.007 and tertiary odds: 0.12, 95%CI: 0.04–0.33, *p* < 0.001), the same factor increases odds of good practice (secondary odds: 3.78, 95%CI: 1.99–7.18, *p* < 0.001 and tertiary odds: 3.78, 95%CI: 1.99–7.18, *p* < 0.001). On the other hand, HPV vaccine knowledge was also very moderate (with majority of mothers not knowing the right age of vaccination; vaccine acceptability was high (90%), but uptake was very low (8% had their daughter vaccinated).

**Conclusion:**

KAP about CC prevention was poor with factors necessary for improvement of KAP identified as education, medical insurance coverage. Making health education easily accessible in schools, primary health facilities and various media platforms will help to address the myths on causes of CC and how it can be treated. Health education and availability of free screening services and free vaccine will improve CC prevention out outcomes.

**Supplementary Information:**

The online version contains supplementary material available at 10.1186/s12905-021-01575-z.

## Background

Cervical cancer (CC) is cancer of the cervix which is caused by persistent infection with high risk human papillomavirus (HPV) [1]. Approximately 19 HPV genotypes are high-risk (HPV16, 18, 26, 31, 33, 35, 39, 45, 51, 52, 53, 56, 58, 59, 66, 68, 70, 73 and 82) [2]. Time from pre-invasive to invasive CC can range from 10 to 20 years that is why screening is a major component of CC prevention [3]. HPV is sexually transmitted [4]. Cervical cancer prevention involves primary and secondary measures depending on the HPV status of the woman. Primary measures include behaviour modification targeting females who have not contracted HPV. Secondary measures involve screening and early detection with treatment of precancerous lesions.

The risk factors for CC include HIV infection, co-infection with other STIs, family history, multiparity, having unprotected sex, number of lifetime sexual partners, age at initial sex activity, current or previous cigarette use, use of oral contraception, current or previous illicit drug use and heavy alcohol intake [5]. The incidence of these factors is disproportionately high in low- and middle-income countries, especially in African countries. Prevention is therefore critical and central to addressing the CC challenge, in such communities where health systems are largely weak due to underfunding, centralisation with majority marginalised interplaying with the high incidence of risk factors.

Cervical cancer is the most common cancer and the leading cause of cancer-related deaths among women in low income countries (LICs) [6]. It is estimated that 300,000 women die from CC every year and that most of them are from poorer LICs [6]. Ways to prevent and treat CC are well known, and death from CC is considered preventable and unnecessary. Even more sobering is the fact that of the 570,000 new cases of CC detected each year globally, almost 85% are from the LICs with highest incidence being reported in East and Southern Africa [7]. Approximately 95% of CC patients are diagnosed in late stage or end stage disease in Africa [8], which can be attributable to the knowledge levels and gaps as well as attitude and practice of all the stakeholders.

The aspect of CC prevention is rapidly evolving because of the positive identification of high-risk HPV genotypes as the major cause of the disease [9]. HPV vaccine benefits include the decrease in incidence of cervical abnormalities, prevalence of vaccine HPV strains and incidence of genital warts [10]. There are no serious safety concerns that are associated with HPV vaccination [11], and the vaccine is an integral component of CC prevention in High Income Countries (HICs) [12]. The best effective vaccination program, if implemented today, would leave a lot of women who are already infected with HPV at risk for CC, hence screening and CC treatment are still critical components of CC control over the coming generation.

HPV vaccine, the bivalent (Gardasil) can provide protection from CC that is caused by HPV-16 and HPV-18 and this makes up 70% of the CC [9]. The quadrivalent (Gardasil 4) vaccine protecting against HPV types 16/18/6/11 and the bivalent (Cervirax) vaccine protecting against infection with HPV-16/18 were both originally used as a three dose schedule [13]. A third vaccine, the Nonavalent HPV (Gardasil 9) vaccine protecting against HPV types 16/18/6/11/31/33/45/52/58 was approved by Food and Drug Administration in 2014 in the USA [13]. Gardasil and Nonavalent also prevent anogenital warts that are caused by HPV 6 and 11 [14]. For LICs, WHO recommends 2 doses for 9 to 14 year old girls and 3 doses for those above 15 years and for those who are immune-compromised [13].

CC is a preventable and curable disease, preventable by vaccination and screening, and curable if identified at an early enough stage [15]. It is gradually becoming a rare disease in many HICs [16]; this is not the case in most low-middle income countries (LMICs) [17]. The incidence rate for CC in Australia was 3.4%, death rate was 1.7% and CC was ranked 24 out of 29 cancers in 2015 [18]. On the other hand, a paper by [19] bemoans the situation in greater parts of Africa and Asia Oceania where invasive CC is comparatively higher and most patients are identified in advanced stages [19]. There was significant under reporting of incidences in African countries, to some extent owing to limited screening and other barriers to seeking medical help [20].

Non-diagnosis is prevalent due to myriad factors, such as financial constraints, cultural and religious beliefs, limited access to services, limited knowledge and lack of education, among other factors, especially in LICs [16]. Furthermore, the other challenge reported in the LICs is the underutilisation of the existing resources in the management of the condition [21]. For example, in the case of Tanzania, [21] found no national guidelines for screening against CC, and where it was done, not all possible medical staff were utilised [21]. According to paper by [21], only 25% of all tertiary institutions in Tanzania were found to perform routine screening, none had a colposcopy and noted that cancer of the cervix tends to be diagnosed in its later stages when it is less treatable in most developing countries [21]. This is despite the fact that forty percent of cancers can be prevented by lifestyle improvement [22]. Such knowledge brings hope to dealing with this disease, and therefore points to the need for better management.

In 2015, Zimbabwe had 7165 new diagnosis of cancer cases and CC was the most common cancer at 19% followed by prostate cancer at 9%; death cases due to cancer were at 2651 of which 1451 were due to CC [23]. Cervical cancer is the leading cause of cancer death among Zimbabwean women and is the commonest cancer among Zimbabwean black women at 34.8% followed by breast cancer at 11.6% [23]. The overall risk of CC increased during the period 1990 to 2010 by a rate of 3.3% annually [24]. For uptake of the vaccine to be optimum, mothers of the young girls need to have knowledge and right attitudes towards the vaccine. There is currently no evidence of mothers KAP, hence this study aims to fill that gap. The aim of the study is to explore knowledge, attitude, practices among mothers of daughters at the vaccination age.

Cervical cancer prevention offers the greatest public health potential and the most cost-effective long-term CC control [26]. Prevention of CC when integrated with other programmes like reproductive health, HIV/AIDs, occupational and environmental health offers the best public long-term method of CC control [26]. Early detection activities are essential in reducing the incidence of CCs in fact, about one-third of the CC burden may be reduced through early detection and treatment of cases at the onset of the disease [27], the phase where treatment is most efficient.

Cervical cancer prevention is more cost-effective than its treatment, therefore it should be the highest priority of any CC control effort. This study hypothesized that: mothers of girls 9 to 14 years lack knowledge about CC prevention, their attitude towards CC prevention is negative and that they are not practicing CC prevention. This study will help to locate where Zimbabwe is on WHO’s strategy to eliminate CC as a public health problem: elimination threshold of 4 cases per 100,000 women, HPV vaccination coverage to 90%, twice-lifetime CC screening to 70%, and treatment of pre-invasive lesions to 90% [28].

## Methodology

### Study area and study settings

The study was conducted in Harare, the capital city of Zimbabwe. Zimbabwe is a LIC located in Southern Africa with a Gross Domestic Product (GDP) per capita of US$908 as of 2016, which is approximately 7% of the world’s average GDP (World Bank national, 2016). Zimbabwe has a population of 16,866,371 people of which 51% of the population are women, and overall dependency ratio is at 52% [24]. Deaths rate is at 178,195 per year, life expectancy is at 49.6 years whilst global life expectancy is at 71 years; 67% of the population lives in the rural areas (United Nations population estimates and projections 2018). Harare has total population of 1,583,902 made up of Males 793,535 (50.1%) and Females 790,367 (49.9%) and estimated annual population growth rate of 2%. The Zimbabwean government rolled out the HPV vaccination programme using school class based model from 14 to 18 May 2018 in Marondera (74 km to the east of Harare, and is capital town of Mashonaland East Province) and Beitbridge (580 km south of Harare at the border with South Africa is the capital town of Matebeleland South) and to the rest of the country in 2019. The study was conducted at Parirenyatwa hospital (a referral hospital in Harare the capital city of Zimbabwe) outpatients department as well as at 20 retail pharmacies in Harare. Zimbabwe is heavily centralised with specialised health services provided primarily from the major cities, Harare and Bulawayo.

### Study design and study population

This cross-sectional study applied mixed methods approach (qualitative and quantitative) with data collected using a semi-structured questionnaire (see Additional file 1). The quantitative and qualitative data was gathered and analysed concurrently- following the embedded approach of mixed methods. Mothers with daughters aged between 9 and 14 years (this is the vaccination age group) were interviewed in assessing their level of knowledge, attitude and practice (KAP) on CC prevention, HPV vaccine as well as the acceptability and uptake of HPV vaccine.

### Sampling technique and sample size

A minimum sample size of 384 mothers was calculated using the Dobson formula with the assumption that distribution of the outcomes being evaluated is at 50% and the desired confidence level is 95% [29]. With the understanding that surveys suffer from low or non-response, incomplete or unusable questionnaires, to help ensure a reasonable sample was achieved, more questionnaires were administered; a total of 420. From that, a total of 406 questionnaires were completed fully and were usable.

A purposive sampling technique was used to select mothers of girls aged between 9 and 14 years. Of the 420 mothers targeted, 206 were sampled from outpatients at different clinics as well as the outpatients’ pharmacy at Parirenyatwa hospital. The other portion of 214 was sampled from 20 selected pharmacies in Harare. The pharmacies were selected from retail pharmacy register kept by Medicines Control Authority of Zimbabwe (MCAZ) using systematic random sampling.

### Recruitment

Inclusion criteria entailed selection of health facilities (one hospital- Parirenyatwa and participating pharmacies from an MCAZ register of pharmacies). From these facilities mothers of daughters aged 9–14 years participated in the study. Neither participants nor their daughters were CC patients but, rather visiting the said health facilities for other reasons- an approach that has been used by [30]. Each woman arriving at the said facilities was approached and informed about the study, and asked to spare time on their way out to participate in the survey, should they be having a daughter of between 9 and 14 years and was willing to respond. The recruitment and surveying took place daily from the time of open until close of the facility (to limit selection bias given possible differences in day and time of the day of visit to health facilities, for example working mothers may visit on weekends or late afternoons) until the target sample was reached. The survey took place between October 2019 and June 2020. All participants were asked to sign a consent form.

The study made use of interviewer-administered paper-based questionnaires. The questionnaires were in three major languages that are spoken in Zimbabwe, that is, English, Shona and Ndebele. The research assistants included 20 pharmacists and 10 nurses who were trained by going through the questionnaire with the principal researcher as well as being briefed on the consent form process according to the Declaration of Helsinki. Mothers who came to the 20 selected pharmacies and to Parirenyatwa hospital outpatients who did not consent to participate in the study were excluded.

### Data management and quality assurance

All questionnaires were numbered, a codebook was created, and data was captured into Microsoft Excel designed data entry template. The process helped to ensure no double entry occurred (each completed questionnaire was uniquely identified) to minimise capturing errors (as codes were captured for quantitative questionnaire items); qualitative data was capture verbatim. In addition, data verification process was possible (Excel template was designed in such a way that every column represent questionnaire item and a heading was written to reflect that), it was also easy to import the data in the analysis software, Stata version 16. A trained data capturer went through all these steps, the process was then verified by the researcher, and then also by the statistician before analysis was done.

The electronic data are backed up on different devices and physical questionnaires are stored in a lockable cupboard with no information linking respondents to the data. Anonymity was ensured in the study by not asking for names of respondents or disclosing them where such information was known by the researcher. Consent forms are kept separately from questionnaires and no information that can link the consent back to questionnaires is available.

A pilot study of 20 participants was done, verifying responses during questionnaire administration, training of research assistants and double-checking during data entry. Data for the pilot interviews was not included in the analysis. Validity and reliability test were also done prior full-scale survey. To ensure validity, responses were double-checked with each respondent and confirmatory factor analysis was used to measured validity objectively. On the other hand, split-half method [31] was used to test reliability of the instrument and to provide confidence for inferences. In addition, any scales in the instrument were subjected to Cronbach Alpha [32] to check reliability, with an alpha of at least 0.70 considered reliable.

### Data analysis

Data was analysed using Stata 16; the data was coded and cleaned before analysis. Descriptive analysis was used to explore distribution of variables as well as identifying the common factors among the responses. Inferential statistics were used to test hypotheses using regression analysis and chi-square test for association as explained below. Logistic regression analysis formed part of the inferential statistics to determine which factors were statistically significant in explaining KAP of mothers of 9–14-year-old girls. Two types of regression analyses were done following the logistic framework, given the non-continuous nature of dependent variables and thus non-linear relationship assumption. Specifically, the following types of logistic regressions were estimated:

1. Ordered logistic regression: This type of regression caters for non-linear associations such as portrayed by a categorical dependent variable and when the outcome is dichotomous as in our case. For this study the interest was to check the predictors of knowing about CC (Knowledge on how the mothers view CC, Attitude e.g. thinking that CC is a disease of the poor, and of screening uptake as revealed Practice), and each of these outcome variables are binary in nature (1 being YES, 0 being No). Separate estimations were done to predict the probability of each of the outcomes.
2. Multinomial logistic regression: the study was also interested in checking the prediction of what patients consider best method of managing CC (preferred than revealed Practice). The outcome variable (what is the best method) is nominal, with 5 categories (it is important to include categories like ‘I don’t know’ and ‘Other’ to capture comprehensively and exhaustively the complete spectrum of what is considered alternative (the interpretation for those who chose any of these categories). Given this nature of outcome variable, multinomial logistic regression was applied.

## Results

The response rate for this survey was 97% (406/420). The mothers surveyed were middle aged, mostly in their youth (mean age of 34 years; SD = 5.18 years), with youngest being 25, and oldest being 48 years of age; while the daughters mean age was 12 years (SD = 1.88, youngest 9, oldest 14). The majority 339 (83.6%) were married; on the other hand, 78 (19.2%) were employed; and 55(13.6%) had medical aid. Slightly more than half, 210 (51.7%) resided in urban; with 21.4% having tertiary education and 404 (99.5%) were Christians. Table 1 is a summary of the socio-demographic profile of the participants.

**Table 1:** Socio-demographic characteristics of the study population (n = 406)

Characteristics/variables	n (%)
Mother’s age: n (%)	
*Mean ± SD (range)*	34 + 5.18
25–30	141(34.8)
31–35	119(29.4)
36–40	101(24.9)
> 40	44(10.9)
Daughter’s age: n (%)	
*Mean ± SD (range)*	12 + 1.88
9	66(16.3)
10	122(30.1)
11	26(6.4)
12	41(10.1)
13	54(13.1)
14	97(24.0)
*Marital status, n (%)*	
Married	339(83.6)
Divorced	36(8.9)
Widowed	31(7.5)
*Employed, n (%)*	
Yes	78(19.2)
No	328(80.9)
*Location, n (%)*	
Urban	210(51.7)
Rural	196(48.3)
*Medical aid, n (%)*	
Yes	55(13.6)
No	355(86.4)
*Education, n (%)*	
Primary	65(16.0)
Secondary	254(62.6)
Tertiary	87(21.4)
*Religion, n (%)*	
Christian	404(99.5)
Traditional	2(0.5)

The demographics presented above were used to interrogate the data further under the KAP analysis presented in the subsections that follow, using cross tabulations (chi-square test of association) and in regression analysis (to determine the prediction power).

### Knowledge of CC

This section presents results from assessing the level of CC and CC prevention knowledge among the respondents as shown in Table 2. Open ended questions such as those seeking definition of CC, causes of CC and definition of HPV were unitised and coded, and then the categories were also summarised quantitatively.

**Table 2:** Knowledge of cervical cancer among mothers of 9–14-year-old girls in Zimbabwe (n = 406)

Variable/question	n (%)
*What is cervical cancer, n (%)*	
Cancer of cervix	292 (71.9)
Abnormal growth of cervical cells	10 (2.5)
Cancer of the uterus	3 (0.7)
Disease found in women's cervix	1 (0.25)
Don’t know	100 (24.6)
*What causes CC (first response), n (%)*	
Witchcraft	234 (57.8)
HPV	128 (31.6)
Miscarriages	39 (9.6)
Any chronic condition	1 (0.3)
Unprotected sex and poor sexual behaviour	1 (0.3)
Don't know	2 (0.5)
*What causes CC (second response), n (%)*	
Vaginal douching	99 (24.2)
HPV	96 (23.7)
Unprotected sex poor sexual behaviour	40 (10.0)
Infection	22 (5.4)
Witchcraft	8 (2.0)
Other	15 (3.9)
Don't know	125 (30.9)
*Can CC be transmitted from person to person, n (%)*	
Yes	107 (26.4)
No	293 (72.4)
Don’t know	5 (1.2)
*Is HPV sexually transmitted infection, n (%)*	
Yes	162 (39.9)
No	224 (55.2)
Don’t know	20 (4.9)
*How many years can you have HPV asymptotically, n (%)*	
10–20	145 (35.8)
Don’t know	255 (63.0)
Other (unspecified)	2 (0.5)
Other-5 years	2 (0.5)
Other- 6 months	1 (0.3)
*Is CC curable?*	
Yes	257 (63.3)
No	145 (35.7)
Don’t know	4 (1.0)

Results in Table 2 show that 100(24.6%) of the mothers did not know what causes CC, 234(57.8%) thought that CC was caused by witchcraft, while 9.6% reported that they think CC is caused by miscarriages. Only 96(23.70%) indicate correctly that it is HPV that causes CC. As respondents were listing more than one causes, on the second one 125(30.9%) of the mothers did not know, and some said ‘vaginal douching’ 99 (24.5%).

Only 24.6% think CC can be sexually transmitted, and 39.9% also think HPV can be sexually transmitted. On the other hand, 224 (55.2%) say HPV cannot be sexually transmitted and 145 (35.7%) said CC is not curable. Majority, 255 (63%) of the mothers did not know how many years one can have HPV and be asymptotic.

In addition, knowing symptoms is central to seeking early medical attention, and this study checked with respondents what they expect to see in CC patients as symptoms (see Additional file 2: Table A1 Symptoms and risk factors). The results show abnormal vaginal bleeding topping the list at 307 (77.3%), followed by unusual discharge from the vagina mentioned by 200(71.0%); then vaginal sores was reported by 234 (58.9%), pain during sex indicated by 232 (58.4%) and weight loss at 225(56.7%) ends the list of the symptoms mentioned by more than 50% of the respondents. On the other hand, the study found the following high risk factors as reported by respondents: immunosuppression 279 (70.6%), HIV 267 (67.6%) and co-infection with other STIs 251 (63.5%).Furthermore, behavioural risk factors were checked, and vaginal douching tops the list 282(71.4%), followed by unprotected sex 277 (70.1%), number of lifetime partners 273 (69.1%), number of STIs treated 236(59.8%) and age at initial sex activity among others.

Knowledge of CC’s association with HIV was assessed and 273 (68.9%) said CC was associated with HIV, 249 (62.9%) said Kaposi Sarcoma and 11(28.8%) said Non-Hodgkin lymphoma (see Additional file 3: Table A2). Furthermore, respondents were asked of the required frequency for CC screening, and majority 238(58.8%) said after every three years; with 193 (47.7%) thinking it is every year, 78(19.3%) consider it to be 5 years while over a third, 140 (34.6%), did not know. The guidelines recommend screening for CC in women age 21 to 65 years with cytology (Pap smear) every 3 years or for women age 30 to 65 years who want to lengthen the screening interval, screening with a combination of cytology and human papillomavirus (HPV) testing every 5 years is required. Over a third of the respondents said they don’t know the required frequency for screening.

In terms of methods used for screening, VIA was reported by 271 (66.8%) and 142(35) said PAP, 99 (25.4%) said HPV while 132 (32.5%) did not know any method of screening*.* Majority, 248(63.4%) has the right knowledge that screening should be done among all women of between 21 and 65 years. Those who reported that screening should be done on all females from birth were 15 (3.8%) and those considering that it must be done by those that have symptoms were 13(3.3%). On the other hand, 9(2.3%) said screening should be done on individuals with family member with CC and only 1 (0.3%) said all breastfeeding mothers.

Besides general understanding of what CC is, and how it should be screened and whom to be screened it is important to know how the disease can be treated.; radiotherapy was the method indicated by most respondents 271(68.6%), while surgery was pointed out by 229 (58%), chemotherapy was identified by 181 (45.8%) and 17 (4.3%) thinks there is no treatment for CC.

Having assessed the knowledge descriptively, inferential analysis was conducted to test which factors explain the knowledge (or lack of) as reported above. To start with, chi-square test for association was done for each of the outcomes and the factors. See Additional file 4: Table A3 presents the summary of test for association results for each of the KAP variable described above.

On what can be done to prevent CC, the responses vary statistically significantly across categories of age (*p* = 0.035), employment (*p* < 0.001), medical aid (*p* < 0.001), resident (*p* = 0.023), and education (*p* < 0.001). Regarding symptoms to be expected in CC patient, the responses differ statistically significantly across categories of marital status, employment, medical aid and education (*p* < 0.001). On the other hand, health risk factors vary statistically significantly across all the demographics variables, age (*p* = 0.012), resident (*p* = 0.008), religion (*p* = 0.001) and *p* < 0.001 for Marital Status, Employment, Medical Aid and Education, while behavioural risk factors are associated with age (*p* = 0.011), location (*p* = 0.012) and *p* < 0.001 for Marital Status, Employment, Medical. Aid, Education and Religion. Responses to cancers considered to be associated with HIV vary statistically significantly across the categories of marital status, employment, medical aid, education (*p* < 0.001) and location (*p* = 0.002); while how often screening need to be done varied statistically significantly across the categories of employment (*p* = 0.005), medical aid (*p* = 0.001), and education (*p* < 0.001). Lastly, method of screening and how CC is treated responses vary statistically significant across the categories’ of all demographic variables (*p* < 0.050) except religion (*p* = 0.902).

Respondents were asked whether they know what CC screening is, answering Yes or No, which helped to measure knowledge. An ordered logistic regression technique was estimated, and the results are presented in Table 3 below. Among the explanatory variables, only age of the respondent is continuous, the others are all categorical. For categorical variables, the first category has been set as reference; therefore, analysis is on what is the probability of knowing about CC screening of category ith (subsequent) compared to category ith − 1 (comparator).

**Table 3:** Logistic regression of factors affecting KAP in CC prevention (n = 406)

Variable	CC knowledge predictors		CC attitude predictors		CC practice predictors	
	Odds ratio [95% CI]	*p*-value	Odds ratio [95% CI]	*p*-value	Odds ratio [95% CI]	*p*-value
Age	1.023(0.95–1.10)	0.536	0.99(0.94–1.03)	0.546	0.98(0.92–1.06)	0.560
*Marital status*						
Married	Ref			0.422		
Divorced	0.59(1.19–1.80)	0.351	1.55 (0.53–4.52)	0.515	0.67(0.24–1.87)	0.441
Widowed	0.11(0.02–0.68)	0.017	0.55(0.09–3.31)		0.95(0.20–4.47)	0.948
*Employed*						
Yes	Ref					
No	0.73(0.31–1.72)	0.469	1.37(0.65–2.92)	0.410	1.20(0.56–2.55)	0.640
*Medical aid*						
Yes	Ref					
No	0.17(0.05–0.59)	0.005	1.23(0.51–2.95)	0.640	0.66 (0.28–1.59)	0.357
*Residential*						
Urban	Ref					
Rural	1.31(0.79–2.19)	0.297	1.35(0.84–2.17)	0.215	0.90(0.57–1.43)	0.653
*Education*						
Primary	Ref					
Secondary	4.20(2.25–7.84)	< 0.001	0.39 (0.20–0.78)	0.007	3.78(1.99–7.18)	< 0.001
Tertiary	7.75(2.04–29.45)	0.003	0.12(.04–0.33)	*p* < 0.001	9.26(3.33–25.70	< 0.001
*Religion*						
Christianity	Ref					
Traditional	0.42(0.022–7.75)	0.557	–		1.24(0.06–24.12)	0.887

Each of the results are discussed under related heading: Knowledge, Attitude and Practice respectively

### Predictors of knowledge

As presented in Table 3, under the columns headed ‘CC knowledge predictors’, association of age with knowledge is not statistically significant; therefore knowledge of CC is not age dependent- the old (up to 48 years in our sample) and the young (as young as 25 years in our sample) have similar level of knowledge on average. The logistic regression used managed to classify 68.1% of the variation in outcome without any explanatory factors, with a log likelihood of 2.13 times for one being knowledgeable (yes on the outcome, than not). The classification increases to 73.5% after controlling for the demographics. Adding explanatory factors result in a model with Chi-square value of 82.57 (*p*-value < 0.001) based on omnibus tests, implying the model and identified factors included are relevant. Overall, the model has a pseudo R^2^ ranging from 0.19 (Cox & Snell) to 0.26 (Nagelkerke) indicating the significant explanatory power of the included factors (demographics) to the outcome variable.

Marital status is significant in explaining CC knowledge as widowed respondents showed lower probability (OR = 0.11, *p* = 0.017) of knowing about CC compared to the married respondents. A person with no medical aid is less likely to know (OR = 0.17, *p* = 0.005) about CC compared to a person with medical aid. The more educated a person is the better is their knowledge about CC, given that the coefficient is even larger and positive at tertiary level (OR = 7.75; *p* = 0.003). The demographics are important in explaining differences in knowledge among mothers of teenage girls in Zimbabwe. Having looked at knowledge, the next section presents results on Attitude and Practice.

### Predictors of attitude

To be able to conduct logistic regression, a question asking respondents whether CC is the disease of the poor or not was re-coded to have: 1 = agree that it is disease of poor (bad/negative attitude) 0 = disagree that is, it is not a disease of the poor (good/positive attitude). In summary, 190 (46.9%) respondents disagree with the statement (had positive attitude, being realistic that CC is a disease affecting rich and poor) and majority 215 (53.1%) disagreed with the statement.

As presented in Table 3 under section “CC Attitude predictors”, only education can turn negative/bad attitude to positive/good, as the results show that those with secondary and tertiary level of education as highest, were less likely to have a negative attitude (0.39 and 0.12 odds ratio respectively).

In addition, Table A5 show the participants’ attitudes towards management of CC. Majority (51.4%) disagreed that herbs help with CC management, while 44% neither agreed or disagreed. About 90% of the participants believe that religion/spirituality helps in managing CC. The highest proportion of 195 (48%) of the respondents were neutral on whether CC is a disease of the poor, with cumulatively193 (47.5%) disagreeing [cumulatively 179 (44%) disagree plus10 (2.5%) strongly disagree].

Attitude reveals behavioural responses to a condition like CC. Behavioural interventions where needed, will target to change attitude first. A cumulative proportion of 120 (29.5%) think that people do suffer from CC due to promiscuity, while 142 (35%) are neutral and a total of at least 142 (35%) disagree. For the four aspects of perspective and practice a significant number of the respondents (average a third) were neutral on these matters. On what is the best way to treat CC; medical approach was identified by the majority 264 (65%), and a quarter stated other; while 25(6.2%) do not know, (10(2.5%) reported herbs and 4(1%) indicated traditional healer.

### Predictors of practice

Of the 406 sample, 226(55.7%) women reported that they have been screened before. For those that have screened, majority of them used VIAC method 162 (71.7%) followed by PAP 60 (26.6%) and lastly HPV 4(1.8%). Demographics are important in CC management as they help understand variation in knowledge, attitude and practice. The study investigated the role of demographics on profiling those who screen, and those who did not screen. Table 3 presents the multiple regression results on columns under ‘CC practice predictors’, showing the odds ratio, 95% CI of the odds ratio and associated *p*-value. The logistic regression managed to classify 55.7% of the variation in outcome without any explanatory factors, with a log likelihood of 1.257 26 times one being knowledgeable (yes on the outcome, than not). The classification increases to 63.6% after controlling for the demographics. Adding explanatory factors result in a model with chi-square value of 46.613 (*p*-value < 0.001) based on omnibus tests, implying the model and identified factors included are relevant. Overall, the model has a pseudo R^2^ ranging from 0.109 11 (Cox & Snell) to 0.146 15 (Nagelkerke) indicating the significant explanatory power of the included factors (demographics) to the outcome variable.

Compared to knowledge which was influenced by marital status, medical aid and education, actual screening is only influenced by level of education as all other factors were insignificant. The higher the level of education, the higher the odds of getting screening (good practice) (one with secondary education is 3.78 times more likely to have been screened than one with primary as highest level; one with tertiary is 9.26 times more likely to have screened for CC than one with primary education as highest level.

In addition, multinomial logistic regression was done (see Table 4 at the end of the paper) on what demographics explain what is considered best way of managing CC by the mothers. This is measuring ‘practice’ as well, given that the most preferred method is the one which will be followed. The dependant variable has multiple categories of nominal in nature, hence the technique applied. Model with predictors show pseudo R-square of 16% (Cox and Snell) to 18.8% (Nagelkerke) and a statistically significant chi-square attesting to the goodness of fit of the model. The reference group is medical (the recommended way of treating CC), the regression then shows which factors predict use of any other method than the medical approach.

**Table 4:** Multinomial logistic regression on factors determining best way of managing CC by mothers (n = 406)

Best Way of managing CC	Coef. (95% CI)	*p*-value
Medical	(base outcome)	
*Herbs*		
AGE	0.20(0.06–0.34)	0.005
*Marital status*		
Divorced	0.99(− 1.49–3.45)	0.437
Widowed	1.12 (− 1.80–4.04)	0.451
*Employed*		
No	− 0.49 (− 2.42–1.44)	0.616
*Medicalaid*		
No	2.54(− 0.31–5.38)	0.08
*Residential*		
Rural	− 2.07 (− 4.33–0.20)	0.074
*Education*		
Secondary	− 1.10 (− 3.06–0.86)	0.272
Primary	− 1.25 (− 4.14–1.64)	0.396
*Traditional healer*		
Age	− 0.06 (− 0.29–0.18)	0.642
*Marital status*		
Divorced	− 12.44 (− 2887.67–2862.80)	0.993
Widowed	3.54 (− 0.18–7.26)	0.062
*Employed*		
No	1.76 (− 4.19–7.70)	0.563
*Medicalaid*		
No	− 0.51 (− 6.14–5.11)	0.858
*Residential*		
Rural	− 0.74 (− 3.15–1.67)	0.548
*Education*		
Secondary	− 1.44 (− 4.00–1.12)	0.27
Primary	− 1.44 (− 6.42–3.54)	0.57
*I_dont_know*		
Age	0.02 (− 0.07–0.10)	0.718
*Marital status*		
Divorced	− 12.57 (− 1047.30–1022.17)	0.981
Widowed	1.50 (− 1.38–4.38)	0.307
*Employed*		
No	1.80 (− 0.58–4.18)	0.139
*Medicalaid*		
No	− 0.19 (− 2.63–2.25	0.878
*Residential*		
Rural	− 1.55 (− 2.71–0.38)	0.009
*Education*		
Secondary	− 1.74 (− 2.80–0.69)	0.001
Primary	− 2.98 (− 5.79–0.17)	0.038
*Any other*		
Age	− 0.03 (− 0.09–0.02)	0.229
*Maritalstatus*		
Divorced	0.99 (− 0.20–2.18)	0.103
Widowed	− 30.88 (− 1.34E+07–1.34E+07)	1.000
*Employed*		
No	0.32 (− 0.63–1.27)	0.51
*Medicalaid*		
No	0.89 (− 0.42–2.20)	0.182
*Residential*		
Rural	0.07 (− 0.47–0.62)	0.792
*Education*		
Secondary	− 1.47 (− 2.15–0.79)	*p* < 0.001
Primary	− 2.85 (− 4.42–1.29)	*p* < 0.001

Results show that the older one is, the more likely (1.23 times more) they believe in herbs compared to medical approach. Those with no medical aid, are less likely (0.07 likely) to mention medical approach than those who have medical aid. Which attest to access and affordability. In addition, those in the rural areas were more likely (4.44 times more) to use herbs than medical approach compared to their counterparts in the urban.

One residing in in rural area is1.36 times more likely not to be knowing which is the best option for treating CC than to indicate medical, compared to their counterparts and those with primary education as the highest level of education are 1.06 times than one without schooling. Those with primary education are 9.36 times more likely to use ‘other’ means for treating cancer than medical compared to those with tertiary education. The overall marginal effects are that the probability of medical method is highest at 0.65, followed by other methods (0.25), not knowing (0.06), herbs (0.03) and traditional healer only (0.01).

The study further interrogated the association between KAP and demographics using cross-tabulation and chi-square tests (See Additional file 4). The results show that, of those who do not know what CC screening is (130), 128 (98.5%) have not been screen of CC. On the other hand, a total of 276(68%) who say they know what CC screening, 81.2% (224) of them screened, while only 52 (18.8%) did not screen). Conversely, the results show that of the 180 that did not screen, 128 (71.1%) did not know what CC screening is and 52 (28.9%) knew but the knowledge did not translate to action (practice). On the other hand, of those that screened for CC, 224 (99.1%) knew what CC screening is. It is therefore apparent that knowledge informs practice and shapes attitude, hence the statistically significant association between the two; Pearson Chi-square = 227 with *p* < 0.001. It addition there is strong association between knowledge of screening, and the practice of actually screening (see Additional file 5).

Having assessed knowledge, attitude and practice of respondents towards CC, the study further explored the mothers understanding and attitude towards the preventative measure of vaccination (see Additional file 6).

### Vaccination

The respondents were asked several questions around vaccination, which they would respond to either Yes, Maybe, No, I don’t know or prefer not to answer. Majority (90.1%) of the mothers support the national vaccination again CC (see Additional file 7). Additionally, they thought the vaccine is a need and they would like to vaccinate their daughters (89.4%). A total of 49.3% believe that HPV vaccine provide protection against CC. About 10% of the mothers reported having their daughter’s vaccinated. When participants asked about the age for vaccination, over 50% reported the right age group for vaccination (19–14 years) and only 47.8% didn’t know which age group. Obstacles to vaccination were explored and included unavailability, religion and cost. School based vaccination programme was cited as the best method followed by use of clinics.

## Discussion

The level of knowledge was found to be poor; the attitude towards CC was negative marred by lack of awareness and some important facts about CC prevention and causes. Screening is at the heart of prevention, early detection and overall management of CC, and is an intricate detail compared to just knowing CC in general. Knowledge of CC screening correlates highly with knowledge of CC in general but mostly not serially correlated with the covariates of CC in general, which makes it a good instrumental variable to proxy CC knowledge (see Additional file 5: Table A4). Practice was not acceptable for majority, evidenced by low screening uptake as well as low HPV vaccine uptake. Practice was hampered by unavailability of screening services and unavailability of HPV vaccine in public institutions. The fears of some mothers were on side effects and efficacy, but HPV vaccine acceptability was high. Most of the mothers recommended school-based strategy, making the vaccine available for free as well as health education and awareness about CC prevention and HPV vaccine.

It was disappointing to note that the most cited cause of CC; vaginal douching, is not empirical. This finding is the same with what was found by [33] in Ghana. Many participants in that study had many misconceptions about the risk factors for CC like having abortions, inserting herbs or other substances into the vagina or exposing the vagina to chemicals caused CC or poor hygiene like washing your vagina with dirty hands [33]. Culturally appropriate CC education interventions are needed aimed at addressing misconceptions and increasing knowledge of CC symptoms and signs as well as their perceived risk of CC and the need for CC screening. Literature such as [27, 34] identified many risk factors which are corroborated with findings here, that literature also highlighted the role of factors such as culture in intermediating the effect of risk factors. Level of knowledge and its correctness determine actions sought to prevent CC, and from where advise and solutions are sought. If one believes CC is caused by witchcraft, then prevention measures maybe sought from witchdoctors and traditional healers, thereby affecting overall uptake and effectiveness of prevention programmes.

VIAC was the popularly mentioned method of screening because it is known to be an effective and affordable screening method and is widely available in Zimbabwe in public hospitals and affordable in private sector (below USD20) [35], which may explain its popularity; PAP is relatively new and some women have reported discomfort with it, and HPV testing can be done after positive results of PAP or on its own. HPV testing has been proven in many studies to be sensitive but it has high infrastructure requirements [36] as a result it is not yet widely available in Zimbabwe hence the mothers did not know it or used it for screening.

Regarding the categorical demographics, and place of residence (urban versus rural) have no effect on knowledge about CC. Zimbabwe is predominantly rural, with majority of population residing in rural areas [37] but during much of the study period there was movement restriction hence the fifty-fifty distribution. Interventions should focus on strengthening knowledge about the disease and how to prevent it in both rural and urban settings.

Availability of husband may help in information gathering, processing and act as a support pillar in decision making. Marital status has been identified as a critical factor in related studies like in [38] where single women had the highest VIAC positives followed by the separated or divorced, with the widowed group having the highest proportion of suspicious of CC lesions. The intervention here would be to offer moral and financial support to single women.

Those with medical aid may find it affordable to go for screening. Such individuals have been identified in this study as having higher probability of knowing about CC compared to those without medical aid. Medical insurance coverage is very low in Zimbabwe, at about 9% [39]; therefore, increase access to medical insurance will have positive effect on CC prevention given the results obtained in this study. In Zimbabwe it has been reported that some medical aid societies subsidise up to 50% of screening costs which may enable some individuals to undergo screening [35]; however, the 50% balance to be borne by the insured may be still prohibitive given the poverty levels in the country hence the need to offer national free or subsidised screening services.

Education is key to knowing and understanding CC matters, the results show that those with secondary education have higher knowledge than those with only primary education as highest level of education. In addition, those with tertiary knowledge had even higher knowledge advantage compared to those with primary education. Most mothers would benefit from written health promotional material as unlike in other studies where this was not possible due to limited literacy [40]. A comprehensive national awareness and education on CC will be the appropriate intervention.

The screening rate is slightly higher than the average baseline (before intervention) screening rates in Africa [41]. Intervention of education in nature mainly, had significant impact in those studies, even up to 60% increase in screening uptake. World Health Organisation (WHO) estimates the screening rate to be 5% LICs, compared to 40–50% in HICs [42].

When the mothers were asked what can be done to improve vaccination uptake, shortage of the vaccine and limited information dissemination were the popular ones, suggesting investing in sourcing the vaccine and that adverts be placed in popular media to unlock uptake. Literature states that HPV prevalence can be decreased significantly when coverage rates of over 50% is achieved by countries [43], in this study 8% said their daughters were vaccinated as compared to an estimate of only 3% having been vaccinated worldwide [44].

This study did the first step to access how much mothers of the girls eligible for HPV vaccine know about HPV so that clear and appropriate messages can be developed for them which will in turn improve CC screening and HPV vaccine uptake. Our data indicate that HPV vaccine awareness is low among the mothers, this result is similar to a study that was done in Thailand, Knowledge regarding the HPV vaccine was quite low, 50% knew the link between HPV and CC whilst 33% knew that the vaccine can be administered to girls prior to become sexually active but vaccine acceptance was 77% if offered for free [45]. For future control of CC, accurate understanding of HPV infection and its relationship to CC is important. Health education targeted to and accessible by the right audiences can reduce health disparities in morbidity and mortality of CC making it possible to eradicate CC. Application of existing CC control knowledge can accelerate progress against CC.

The main reason for poor HPV vaccine uptake found in this study was the unavailability of the vaccine. In literature reasons for poor uptake of HPV included factors such as cost, unavailability, poor financing mechanisms, inadequate health system capabilities, cold-chain constraints and low prioritisation of adolescent vaccination [46]. Vaccine hesitancy [46] was not a problem in this study since 89% indicated that they would want their daughters to be vaccinated.

Improved awareness campaigns and use of role models may improve knowledge and demystify some negative beliefs. Wrong information such as role of witchcraft, poverty as a determining factor causes unnecessary fears and anxieties on mothers creating a burden for prevention and management of CC. Majority of women are religious, it is imperative to inform and educate religious leaders, especially women during their own religious gatherings to promote open discussion and create a network of support.

The attitude towards CC prevention was negative with some mythical believes such as some mothers believing that CC infection affects the poor only, is caused by witchcraft, doubting the efficacy of the vaccine among other wrong beliefs. Some even considered it incurable, or a curse; implying that such individuals will not seek medical help. CC survivor’s and those living with the disease need to be role models used in campaigns sharing their experiences. Education through social networks like religious groups and some formal set-ups as in schools may help to have positive attitude towards CC prevention. Subjects such as General Science, Biology, and Family & Heritage need to contain CC content, just as has been the case with Malaria, Cholera, HIV-AIDS, STIs, and other conditions. The curriculum needs to speak to pertinent issues like CC prevention. For example, Malaria is studied from the lifecycle of a mosquito; due to how it was prominent then- the same approach is needed towards CC to win the battle.

Knowledge and attitude determine practice; in some instances, practices are often hampered by unavailability of the services. Even though there is knowledge and willingness of taking vaccine and screening; uptake has been prevented by costs and general unavailability of services. Government, private sector and non-governmental organisation should converge efforts to provide free vaccine across the country and ensuring screening is accessible to all. Investment in health sector is paramount, infrastructure, human capital and vaccines- a health population is productive as there is less absenteeism. For adult women, screening should be combined with other health check-ups or visits such as for family planning, antenatal and postnatal clinics programmes to be available at all primary health facilities in the country. Prevention reduces pressure of care which often requires expensive infrastructure and other resources. With such widespread services, it becomes a norm that when a girl turns 9 years she can be taken in for vaccination just as how citizens have found it normal to go for BCG/Polio vaccination as part of ‘baby’ clinics. Knowledge and attitude will help normalise the practice and improve uptake, especially when resources are deployed adequately. More effort is needed to educate mothers about CC prevention and to make HPV vaccine available.

Social desirability bias is a limiting factor in this study just like in any interviewer administered questionnaire study. This study was a health institution-based study including a selected population of women who could be already having a positive attitude towards their health according to the health belief model [47].

## Conclusion

Women at risk of developing CC need accurate information to understand prevention methods and to enable them to use screening services and to vaccinate their daughters against HPV. Knowledge of the mothers about CC is elementary at its best and there was a high prevalence of ignorance about prevention, causes and risk factors for CC. The uptake of prevention was hampered by unavailability of the CC prevention services. HPV vaccine is acceptable as a prevention method, but uptake is determined by availability of the free vaccine and obtaining appropriate information about the vaccine. A lot of effort should be made to educate mothers about CC prevention and to make HPV vaccine available as well as screening services in public institutions.

## Supplementary Information

**Additional file 1**: Instrument- The questionnaire.

**Additional file 2**: CC Symptoms and risk factors .

**Additional file 3**: Cancer prevention and cancers associated with HIV.

**Additional file 4**: Association between KAP variables and demographics.

**Additional file 5**: Association between knowledge and Practice.

**Additional file 6**: Attitude and Practice frequency distribution.

**Additional file 7**: Vaccination related questions.

## Data Availability

The datasets and materials used in this study are available on request from the corresponding author.

## Abbreviations

AIDS: Acquired immunodeficiency syndrome
BREC: Biomedical Research Ethics Committee
CC: Cervical cancer
CIN: Cervical intraepithelial neoplasia
GDP: Gross domestic product
HICs: High income countries
HIV: Human immunodeficiency virus
HPV: Human papillomavirus
IQR: Inter-quartile range
KAP: Knowledge, attitude and practice
LICs: Low income countries
LMICs: Low- and middle-income countries
MCAZ: Medicines control authority of Zimbabwe
MRCZ: Medical research council of Zimbabwe
PAP: Papanicolaou
SD: Standard deviation
SSA: Sub-Saharan Africa
VIAC: Visual inspection with acetic acid and camera
WHO: World Health Organisation
