# Supplementary material for: Knowledge, attitude and practice towards cervical cancer prevention among mothers of girls aged between 9 and 14 years: a cross sectional survey in Zimbabwe
Source: BMC Womens Health. 2021 Dec 20;21:426. doi: 10.1186/s12905-021-01575-z (PMC8691087; doi:10.1186/s12905-021-01575-z)
Supplement: Supplementary file 1 — Additional file 1: Instrument- The questionnaire. [file 12905_2021_1575_MOESM1_ESM.docx]

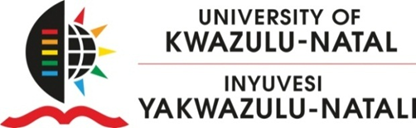


**Study title: Knowledge, Attitude and Practice towards Cervical Cancer Prevention among Mothers of girls aged between 9 and 14 years: a cross sectional survey in Zimbabwe**

**BREC Approval No.:05819**

**MRCZ Approval No:A2505**

**Questionnaire No.**  [ ][ ][ ]

**Study site ID number:** [ ][ ][ ] **Date:** [ ][ ] / [ ][ ] / [ ][ ][ ][ ]

dd mm yyyy

**Instructions**

1. **All participants should be mothers of girls aged between 9 and 14 years**
2. **This questionnaire is pre-coded, please mark clear with cross (X) in the space provided.**

**A. SOCIO-DEMOGRAPHICS**

1. Age
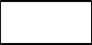
 Marital status: Single
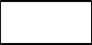
 Married
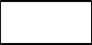


Employed: Yes
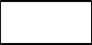
 No
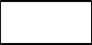
 Medical aid Yes
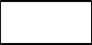
 NO
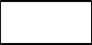


Residential: Urban
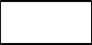
 Rural
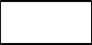


Educational levels: Primary
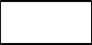
 Secondary
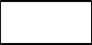
 Tertiary
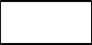


What is your religon? Christian
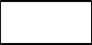
 Muslim
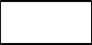
 Traditional
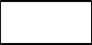
 Other.....................

What is the age of your daughter?
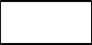


**B. KNOWLEDGE**

2. What is cervical cancer?....................................................................................................................

3. What causes cervical cancer?...................................................................................................................

4. Can cervical cancer be transmitted from one person to another? Yes
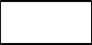
 No
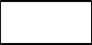


5. How is cervical cancer transmitted?.............................................................................................................................

6. Is cervical cancer a curable Yes
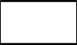
 No
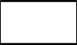


7. What can be done to prevent one from getting cervical cancer?

Don’t know
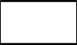
 Vaccine
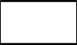
 Avoid teenage sexual activity
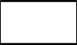


Use of condoms
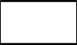
 Good sexual behaving
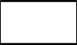
 other…………………………................................

8. What do you think should be done so that people exercise measures that prevent them from contracting cervical cancer?............................................................................................................................

9. What do you think cause people to have cervical cancer?........................................................................

10. What symptoms do you expect to see when someone is having cervical cancer?

a) Abnormal vaginal bleeding
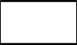
 b) An unusual discharge from the vagina
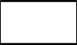


c) Pain during sex (vaginal intercourse)
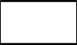
 d) Changes to bowel and bladder habits
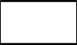


e) Blood in your urine  
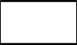
 f) Bone pain
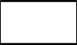
 g) Tiredness and lack of energy
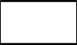


h) Weight loss
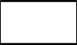
 i) I don’t know
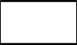


j) Others ………………………………………………………….

11. What are the biological risk factors of cervical cancer?

a) Immunosuppression
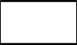
 b) Co-infection with other STIs
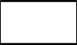
 c) HIV infection
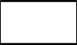


d) Type of HPV infection
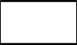
 e) Co-infection with multiple HPV types
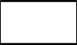


f ) Viral load
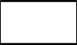
 g) I don’t know
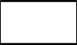
 i) Others................................................................................

12. What are the behavioural risk factors of cervical cancer?

a) Lifetime number of sexual partners
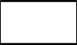
 b) Pattern of condom use
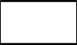


c) Age at exposure to HPV Parity
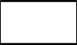
 d) marital status
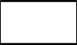


e) Heavy alcohol use
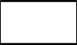
 f ) Current or previous cigarette use
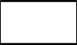


g) Current or previous illicit drug use
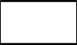
 f) I don’t know
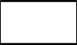
 others ........................................

13. What can an individual do to prevent cervical cancer**?**………………………………………………………………………………………

14. Which cancers are associated with HIV?

Kaposi Sarcoma Cervical

Non-Hodgkin lymphoma
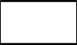
 don’t know
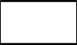
 Other................................................

15. How often should one get screened for cervical cancer?

Six months
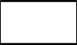
one year
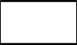
 Two years
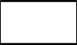
 don’t know
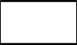
 other …………………..

16. What method is used to screen for cervical cancer? PAP
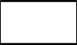
 VIAC
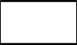
 HPV
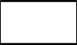
Don’t Know
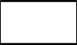
 Other..............

17 How is cervical cancer treated? Surgery
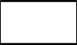
 Radiotherapy
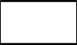
 Chemotherapy
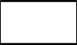
 Don’t Know
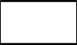
Other...............

18. What other facts do you know about Cervical Cancer?...........................................................................

19. What do you think should be done to improve people’s knowledge of cervical cancer?.............................................

..............................................................................................................................................................................................

**C.PRACTICE AND ATTITUDE**

20. Have you been screened for cervical cancer? Yes
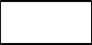
 No
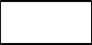


21. Do herbs help in the management of cervical cancer? Strongly Agree
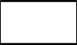
 Agree
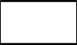
 Neutral
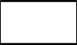
 Disagree
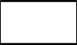
 Strongly Disagree
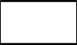


22. What’s your opinion on herbal medicines for cervical cancer treatment?....................................................... ........................................................................................................................................................................

23. Does religion help in the management of cervical cancer? Strongly Agree
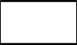
 Agree
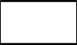
 Neutral
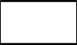
 Disagree
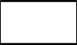
 Strongly Disagree
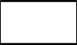


24.Whats your opinion on the importance of religion on cervical cancer management ?..................................

...................................................................................................................................................................................

25.Is cervical cancer a disease of the poor?Strongly Agree
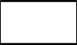
 Agree
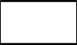
 Neutral
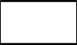
 Disagree
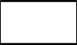
 Strongly Disagree
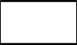


Why..............................................................................................................................................................................

...........................................................................................................................................................................................

26.Are people who suffer from cervical cancer promiscus?Strongly Agree
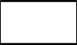
 Agree
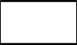
 Neutral
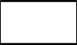
 Disagree
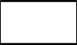
 Strongly Disagree
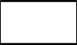
Why...............................................................................................................................................................................

.........................................................................................................................................................................................

27. Would you like to say something about cervical cancer management?..............................

........................................................................................................................................................................

28. Where do you get information about cervical Cancer?**..............................................................................**

**D.Vaccination**

29. Have you ever had about cervical cancer vaccine? Yes
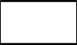
 No
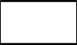


30. Who should be vaccinated? Don’t know
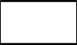
 Girls 9 to 14yrs
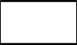
 other................................

31. Have your daughter been vaccinated against cervical cancer? Yes
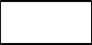
 No
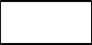


If no, what are the reasons? .......................................................................................

32. Do you support national vaccination for cervical cancer? Strongly Agree
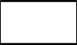
 Agree
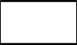
Neutral
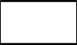
 Disagree
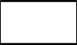
 Strongly Disagree
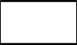


What are your reasons for or against?.........................................................................................................

.....................................................................................................................................................................

33. How should the government go about the national vaccination?......................................................................

..........................................................................................................................................................................

34. What are challenges to anticipate in such a national cervical cancer vaccination? ...........................................

............................................................................................................................................................................

35. How can such challenges be overcome?...................................................................................................................

....................................................................................................................................................................................

36. Are you comfortable with someone close to you receiving the vaccination? Strongly Agree
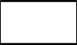
 Agree
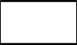
 Neutral
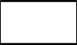
 Disagree Strongly Disagree

What are your reasons for the reservation with cervical cancer vaccination?............................................................

.....................................................................................................................................................................................

**END OF QUESTIONNAIRE**

**THANK YOU**
