# Supplementary material for: Knowledge, attitude and practice towards cervical cancer prevention among mothers of girls aged between 9 and 14 years: a cross sectional survey in Zimbabwe
Source: BMC Womens Health. 2021 Dec 20;21:426. doi: 10.1186/s12905-021-01575-z (PMC8691087; doi:10.1186/s12905-021-01575-z)
Supplement: Supplementary file 2 — Additional file 2: CC Symptoms and risk factors . [file 12905_2021_1575_MOESM2_ESM.docx]

**Additional File 2- Symptoms and risk factors**

**Table A1: Symptoms to expect to see in CC patients and risk Factors**

| **Symptoms** | **n(%*)** | |
| --- | --- | --- |
| Abnormal vaginal bleeding | 307(77.3) | |
| An unusual discharge from the vagina | 282(71.0) | |
| Vaginal sores | 234(58.9) | |
| Pain during sex (vaginal intercourse) | 232(58.4) | |
| Weight loss | 225(56.7) | |
| Bone pain | 173(43.6) | |
| Tiredness and lack of energy | 157(39.6) | |
| Blood in your urine | 120(30.2) | |
| Changes to bowel and bladder habits | 113(28.5) | |
| **Health risk factors** |  | |
| Immunosuppression | 279 (70.6) | |
| HIV infection | 267(67.6) | |
| Co-infection with other STIs | 251(63.5) | |
| Family history | 142(36.0) | |
| Multiparity | 135 (34.2) | |
| Type of HPV infection | 134(33.9) | |
| High viral load | 115(29.1) | |
| **Behavioural risk factors** |  | |
| Vaginal douching | 282(71.4) |  |
| Having unprotected sex | 277(70.1) |  |
| Number of lifetime sexual partners | 273(69.1) |  |
| Number of STIs treated | 236(59.8) |  |
| Age at initial sex activity | 223(56.5) |  |
| Current of previous cigarette use | 157(39.8) |  |
| Use of oral contraception | 154(39.0) |  |
| Current of previous illicit drug use | 134(33.9) |  |
| Heavy alcohol intake | 130(32.9) |  |

** Multiple response variable percent sum > 100*
