# Supplementary material for: Knowledge, attitude and practice towards cervical cancer prevention among mothers of girls aged between 9 and 14 years: a cross sectional survey in Zimbabwe
Source: BMC Womens Health. 2021 Dec 20;21:426. doi: 10.1186/s12905-021-01575-z (PMC8691087; doi:10.1186/s12905-021-01575-z)
Supplement: Supplementary file 3 — Additional file 3: Cancer prevention and cancers associated with HIV. [file 12905_2021_1575_MOESM3_ESM.docx]

**Additional File 3**

**Table A2: Prevention of CC and the cancers associated with HIV**

| **What can be done to prevent one from getting CC** | |  |  |
| --- | --- | --- | --- |
|  | **Frequency** | **Percent of responses** | **Percent of cases** |
| Vaccine | 128 | 34.69 | 44.44 |
| Avoid teenager sex | 161 | 43.63 | 55.90 |
| Use condoms | 41 | 11.11 | 14.24 |
| Good sexual behaviour | 39 | 10.57 | 13.54 |
| **Total** | **369** | **100.00** | **128.13** |
| **Cancers are associated with HIV** | |  |  |
| Kaposi Sarcoma | 249 | 39.15 | 62.88 |
| Cervical | 273 | 42.92 | 68.94 |
| Non-Hodgkin lymphoma | 114 | 17.92 | 28.79 |
| **Total** | **636** | **100.00** | **160.61** |
