# Supplementary material for: Knowledge, attitude and practice towards cervical cancer prevention among mothers of girls aged between 9 and 14 years: a cross sectional survey in Zimbabwe
Source: BMC Womens Health. 2021 Dec 20;21:426. doi: 10.1186/s12905-021-01575-z (PMC8691087; doi:10.1186/s12905-021-01575-z)
Supplement: Supplementary file 4 — Additional file 4: Association between KAP variables and demographics. [file 12905_2021_1575_MOESM4_ESM.docx]

**Additional File 4**

**Table A3: Association between KAP variables and demographics**

|  | Associated factor. *order of cell content*: $\left[ \begin{matrix} {Chi}^{2} \\ P-value \end{matrix} \right]$ | | | | | | |
| --- | --- | --- | --- | --- | --- | --- | --- |
| KAP variable | Age | Marital Status | Employment | Med. Aid | Resident | Education | Religion |
| What can be done to prevent CC | 26.800*  0.035 | 7.123  0.714 | 68.212***  0.000 | 65.671***  0.000 | 13.000*  0.023 | 97.954***  0.000 | 7.254  0.202 |
| Symptoms | 184.620  0.141 | 180.202***  0.000 | 121.875***  0.000 | 137.426***  0.000 | 72.513  0.057 | 198.048***  0.000 | 7.545  1.000 |
| Health risk factors | 140.412*  0.012 | 172.306***  0.000 | 114.252***  0.000 | 118.119***  0.000 | 58.070**  0.008 | 178.500***  0.000 | 65.931**  0.001 |
| Behavioural risk factors | 161.956*  0.011 | 294.237***  0.000 | 107.134***  0.000 | 126.176***  0.000 | 64.219*  0.012 | 205.377***  0.000 | 198.058***  0.000 |
| Cancers associated with HIV | 34.087  0.083 | 42.371***  0.000 | 64.900***  0.000 | 76.470***  0.000 | 24.553**  0.002 | 134.327***  0.000 | 1.868  0.985 |
| How often screening | 18.532  1.000 | 6.116  0.634 | 15.092***  0.005 | 18.509**  0.001 | 9.362  0.053 | 32.618***  0.000 | 1.767  0.778 |
| Method of screening | 30.126*  0.011 | 23.643**  0.009 | 82.593***  0.000 | 104.620***  0.000 | 21.501**  0.001 | 120.027***  0.000 | 1.596  0.902 |
| How is CC treated | 56.160*  0.037 | 44.389**  0.010 | 92.177***  0.000 | 99.176***  0.000 | 25.420*  0.020 | 152.570***  0.000 | 1.088  1.000 |

*Values are statistically significant P<0.05* P<0.01** P<0.001****
