# Supplementary material for: Knowledge, attitude and practice towards cervical cancer prevention among mothers of girls aged between 9 and 14 years: a cross sectional survey in Zimbabwe
Source: BMC Womens Health. 2021 Dec 20;21:426. doi: 10.1186/s12905-021-01575-z (PMC8691087; doi:10.1186/s12905-021-01575-z)
Supplement: Supplementary file 5 — Additional file 5: Association between knowledge and Practice. [file 12905_2021_1575_MOESM5_ESM.docx]

**Additional File 35-** **Association between knowledge and Practice**

**Table A4: Association between knowledge and Practice**

|  | **Have you been screened of CC?** | | |
| --- | --- | --- | --- |
| **Do you know what CC screening is?** | **No** | **Yes** | **Total** |
| **No** | 128 | 2 | 130 |
|  | 98.5 | 1.5 | 100.00 |
|  | 71.1 | 0.9 | 32.02 |
| **Yes** | 52 | 224 | 276 |
|  | 18.8 | 81.2 | 100.00 |
|  | 28.9 | 99.1 | 67.98 |
| **Total** | 180 | 226 | 406 |
|  | 44.3 | 55.7 | 100.00 |
|  | 100.00 | 100.00 | 100.00 |

Pearson chi2(1) = 227.0138 Pr = 0.000
