# Supplementary material for: Knowledge, attitude and practice towards cervical cancer prevention among mothers of girls aged between 9 and 14 years: a cross sectional survey in Zimbabwe
Source: BMC Womens Health. 2021 Dec 20;21:426. doi: 10.1186/s12905-021-01575-z (PMC8691087; doi:10.1186/s12905-021-01575-z)
Supplement: Supplementary file 6 — Additional file 6: Attitude and Practice frequency distribution. [file 12905_2021_1575_MOESM6_ESM.docx]

**Additional File 6- Attitude and Practice frequency distribution**

**Table A5: Practice and Attitude**

| **Variable** | **Category** | **Freq.** | **Percent** |
| --- | --- | --- | --- |
| **Do herbs help in management of CC?** |  |  |  |
|  | Strongly agree | 6 | 1.5 |
|  | Agree | 13 | 3.2 |
|  | Neutral | 180 | 44.3 |
|  | Disagree | 185 | 45.6 |
|  | Strongly disagree | 22 | 5.4 |
| **Does religion help in management of CC?** |  |  |  |
|  | Strongly agree | 127 | 31.3 |
|  | Agree | 241 | 59.4 |
|  | Neutral | 37 | 9.1 |
|  | Disagree | 1 | 0.3 |
| **Is CC a disease of the poor?** |  |  |  |
|  | Agree | 19 | 4.7 |
|  | Neutral | 196 | 48.4 |
|  | Disagree | 180 | 44.4 |
|  | Strongly disagree | 10 | 2.5 |
| **Do people suffer from CC due to promiscuity?** |  |  |  |
|  | Strongly agree | 6 | 1.5 |
|  | Agree | 114 | 28.4 |
|  | Neutral | 140 | 34.8 |
|  | Disagree | 138 | 34.3 |
|  | Strongly disagree | 4 | 1 |
| **What is the best way of treating CC?** |  |  |  |
|  | Medical | 262 | 64.9 |
|  | Herbs | 10 | 2.5 |
|  | Traditional healer | 4 | 1.0 |
|  | I don’t know | 25 | 6.2 |
|  | other | 103 | 25.5 |
