# Supplementary material for: Knowledge, attitude and practice towards cervical cancer prevention among mothers of girls aged between 9 and 14 years: a cross sectional survey in Zimbabwe
Source: BMC Womens Health. 2021 Dec 20;21:426. doi: 10.1186/s12905-021-01575-z (PMC8691087; doi:10.1186/s12905-021-01575-z)
Supplement: Supplementary file 7 — Additional file 7: Vaccination related questions. [file 12905_2021_1575_MOESM7_ESM.docx]

**Additional File 7- Vaccination**

**Table A5:** **Vaccination related questions (yes response)**

| **Question** | **Frequency** | **Percent** |
| --- | --- | --- |
| Is cervical cancer preventable? | 238 | 58.9 |
| Have you ever heard about HPV vaccine? | 208 | 51.5 |
| Does HPV vaccine provide protection against CC? | 199 | 49.3 |
| Has your daughter been vaccinated against CC? | 32 | 8.3 |
| Do you support national vaccination of CC? | 364 | 90.1 |
| Do you think HPV vaccine is needed? | 361 | 89.4 |
| Would you like your daughter to be vaccinated? | 361 | 89.4 |
